# Analysis of mRNA deadenylation by multi-protein complexes

**DOI:** 10.1016/j.ymeth.2017.06.009

**Published:** 2017-08-15

**Authors:** Michael W. Webster, James A.W. Stowell, Terence T.L. Tang, Lori A. Passmore

**Affiliations:** MRC Laboratory of Molecular Biology, Cambridge, UK

**Keywords:** 5- or 6-FAM, 5- or 6-carboxyfluorescein, ARE, AU-rich element, EMSA, electrophoretic mobility shift assay, PAGE, polyacrylamide gel electrophoresis, TBE, Tris-Borate-EDTA, UTR, untranslated region, Poly(A) tail, Gene expression, Exonuclease, RNA, Ccr4-Not, Pan2-Pan3

## Abstract

Poly(A) tails are found at the 3′ end of almost every eukaryotic mRNA and are important for the stability of mRNAs and their translation into proteins. Thus, removal of the poly(A) tail, a process called deadenylation, is critical for regulation of gene expression. Most deadenylation enzymes are components of large multi-protein complexes. Here, we describe an *in vitro* deadenylation assay developed to study the exonucleolytic activities of the multi-protein Ccr4-Not and Pan2-Pan3 complexes. We discuss how this assay can be used with short synthetic RNAs, as well as longer RNA substrates generated using *in vitro* transcription. Importantly, quantitation of the reactions allows detailed analyses of deadenylation in the presence and absence of accessory factors, leading to new insights into targeted mRNA decay.

## Highlights

• An *in vitro* assay for multi-subunit deadenylation enzymes is described.
• Quantitation allows determination of deadenylation rate.
• The effects of accessory proteins on rate and processivity can be measured.

## Graphical abstract

**Figure:**
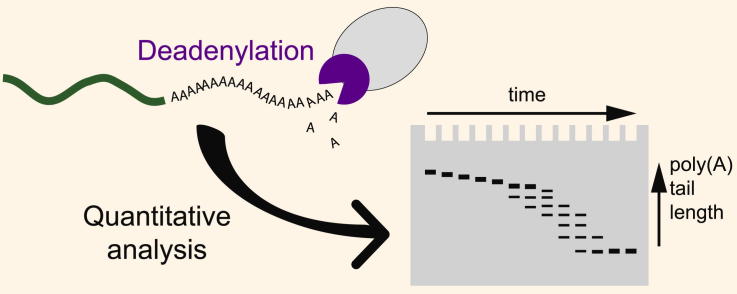

## 1 Introduction

Gene expression levels depend on rates of mRNA turnover. Removal of the poly(A) tail from an mRNA, a process called deadenylation, is a critical and conserved process in eukaryotic mRNA decay [1]. It is the first step in general mRNA turnover, and is often rate-limiting in this pathway [2]. Deadenylation can also be triggered during mRNA quality control [3] and miRNA-mediated repression of gene expression [4]. The rate of poly(A) tail removal is a strong indicator of the half-life of an mRNA, with unstable transcripts undergoing more rapid deadenylation [2]. The rate that a particular mRNA undergoes decay can change and is regulated, for example, in response to environmental signals [5]. The regulation of deadenylation is therefore fundamental for cellular maintenance, but also allows responses to cellular cues. In addition to its role in RNA decay, the removal of the poly(A) tail is also linked to translation repression. This is partly because it coincides with the release of poly(A)-binding protein (Pab1/PABPC), which is required for efficient translation initiation [6].

The poly(A) tail is shortened in the 3′ to 5′ direction by the activity of adenosine-selective exonuclease enzymes. Several proteins have been identified that catalyze this reaction. The most highly conserved are Ccr4/CNOT6, Caf1/CNOT7, and Pan2 [7]. Importantly, these enzymes do not act as monomeric proteins, but are components of the larger multi-protein assemblies Ccr4-Not and Pan2-Pan3. Additionally, other factors, such as RNA-binding proteins and miRNAs, can recruit these deadenylase complexes to specific mRNA transcripts, promoting their deadenylation (e.g. [8], [9], [10], [11]). By controlling mRNA stability in a transcript-specific manner, these proteins thereby regulate gene expression.

Given the central role of deadenylation in post-transcriptional regulation of gene expression, factors that control its rate are of great importance to many biological processes. For example, the control of the cell cycle, embryogenesis and the inflammatory response are known to depend on targeted deadenylation activity [8], [12], [13]. The network of proteins that orchestrate this activity is highly complex, and this has limited our understanding of its mechanism. Studies into the phenotypes of gene deletions have yielded many key insights, but genetic approaches are frequently hampered by compensatory mechanisms within the cell. For example, in the absence of efficient deadenylation, gene expression is repressed using alternative mechanisms, such as decreased transcription. This compensates for impaired mRNA decay, buffering the mRNA and protein quantities in the cell [14]. Therefore, even though deadenylation is the first step of general mRNA decay, deletion of genes encoding the nucleases of Ccr4-Not is not lethal in yeast [15], likely because overall mRNA levels remain relatively unaffected. In contrast, deletion of the poly(A) binding protein Pab1, or the Not1 subunit of Ccr4-Not is lethal [16], [17]. This has made it challenging to dissect the specific functions of these proteins *in vivo*. Finally, the proteins involved in mRNA decay often play multiple roles in this process. For example, Ccr4-Not and its accessory factors can inhibit gene expression by catalyzing deadenylation, recruiting decapping stimulators [18], [19], inhibiting translation [20], [21], and regulating mRNA localization [22]. In many cell-based experiments, the contribution of each of these various mechanisms is consequently unclear.

For these reasons, it is advantageous to directly characterize deadenylation using an *in vitro* system reconstituted from purified factors. Previously, the activities of individual nuclease enzymes of Ccr4-Not have been examined [15], [23], [24], [25]. Since both Ccr4-Not and Pan2-Pan3 are thought to be obligate multi-protein complexes in the cell, it is unclear whether the activities of isolated nucleases are representative of their activities within the context of their complexes. Furthermore, targeted deadenylation of specific mRNAs is typically a property of the intact complexes. This is because the RNA-binding adaptor proteins that link Ccr4-Not to a particular set of mRNAs (e.g. Tristetraprolin, Nanos, Roquin, and GW182) typically bind to the non-enzymatic subunits of Ccr4-Not [26], [27], [28], [29]. The recruitment of Pan2-Pan3 to RNA is likewise believed to be mediated by interactions between the non-enzymatic Pan3 subunit and Pab1 [30]. Therefore, assays of deadenylation activity are best performed using intact complexes.

We recently reported methods for the purification of recombinant Pan2-Pan3 [31] and Ccr4-Not [32]. These complexes can also be prepared from endogenous sources [9], but lower yields prevent rigorous purification and subsequent quantitative *in vitro* analyses. The larger quantities obtained from a recombinant system permit more stringent purification, which minimizes co-purification with contaminating RNases and endogenous factors that may regulate the deadenylation activity. Furthermore, we have found that the constituent subunits of recombinant protein complexes are purified in stoichiometric ratios that likely match the physiological compositions [31], [32].

Here, we describe a method for analyzing the deadenylation activities of the Ccr4-Not and Pan2-Pan3 deadenylation complexes *in vitro*. By examining reactions performed with polyadenylated RNA substrates of uniform length, the sequential removal of adenosine nucleotides can be visualized at high resolution by denaturing polyacrylamide gel electrophoresis. This allows quantitation of the reaction rate. We provide a detailed rationale for the experimental setup, as well as key considerations necessary to obtain reliable and accurate results. While the deadenylation activity of Ccr4-Not is shown here as an example, the techniques described could aid in high-resolution analysis of the RNase or DNase activities of a variety of other enzymes.

## 2 Assay overview

**Fig. 1:**
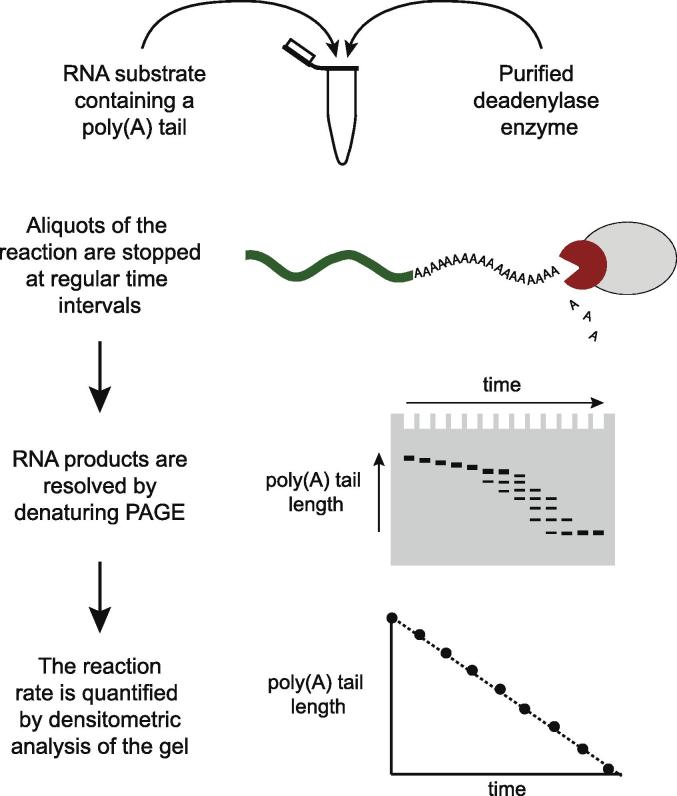
Workflow for the *in vitro* measurement of deadenylation activity of purified proteins. Deadenylation assays are initiated by addition of purified deadenylase protein to an RNA substrate containing a poly(A) tail. The RNA can be generated by chemical synthesis or *in vitro* transcription. During incubation, aliquots of the reaction are removed at regular time intervals and stopped by the addition of denaturing loading dye. The RNA products are resolved by denaturing polyacrylamide gel electrophoresis. The reaction is quantified by densitometric analysis of the gel lanes.

A minimal deadenylation reaction contains three components: polyadenylated RNA substrate, purified deadenylase enzyme, and a buffer solution. We designed such a system to examine the deadenylation activities of Ccr4-Not and Pan2-Pan3 *in vitro* (Fig. 1) [31], [32]. Briefly, deadenylation enzymes are incubated with a purified RNA substrate containing a poly(A) tail of defined length. Samples are taken at regular intervals and the poly(A) tail length is analyzed by denaturing gel electrophoresis. Quantitation of the gel allows evaluation of the rate of deadenylation, the processive vs. distributive nature of the enzyme, and the effect of accessory proteins on activity. Details of each of these steps are discussed below.

## 3 Design of RNA substrates for deadenylation

A variety of RNA substrates can be used in assays to investigate the rates and patterns of deadenylation. Here we evaluate the types of RNAs used in previous studies, and highlight the advantages of each.

### 3.1 Short synthetic RNAs

Since deadenylation is the exonucleolytic removal of adenosines from the 3′ end of RNA, the simplest substrate is a short polyadenosine RNA such as A10 or A15 [24], [33]. Specificity for the removal of adenosines can be assessed by comparing these reactions with those performed with other RNA homopolymers: poly(C), poly(U) and poly(G) [24], [34]. RNA substrates containing poly(G) tracts can form G-quadruplexes, which may hinder or complicate analysis of deadenylase specificity [35].

To examine whether exonuclease activity is limited to the poly(A) tail, substrates containing ∼20 non-poly(A) nucleotides upstream of 10–30 contiguous adenosines have been used [23], [25], [36], [37], [38]. These studies revealed that deadenylase enzymes display specificity for nucleotides on both sides of the scissile bond [36], [39], and as a result, the adenosine proximal to a 3′ UTR may not be rapidly removed [23], [37]. In addition, the use of longer RNAs may promote binding of the deadenylase enzymes to the RNA substrate [23]. We have found this type of model substrate to be highly versatile. As they are shorter than ∼50 nucleotides in length, they can be commercially synthesized with reasonable yield, purity and affordability [40]. The products of the deadenylation reaction can be separated by polyacrylamide gel electrophoresis at single-nucleotide resolution due to their small size (see Section 6).

Furthermore, sequence motifs can be incorporated into the upstream non-poly(A) sequence. RNA-binding proteins that regulate deadenylation typically bind to short sequence motifs 4–10 nucleotides in length. The sequences bound by many of these proteins have been defined [41], [42], [43], and RNA substrates of the type N_20_A_10-30_ can be commercially synthesized to reconstitute and characterize targeted deadenylation *in vitro*. For example, by adding a specific mRNA destabilizing element (the ‘determinant of selective removal’ or DSR; UUAAAC) to a short synthetic RNA with an A30 tail, we could reconstitute targeted deadenylation by Ccr4-Not with the adaptor protein Mmi1 [32]. Deadenylation is also accelerated by miRNAs. The seed sequence by which miRNAs recognize mRNA targets is typically 6–8 nucleotides in animals [44]. In principle, short polyadenylated RNA substrates could therefore also be used to study miRNA-mediated stimulation of deadenylation.

### 3.2 Longer *in vitro* transcribed RNAs

While short model substrates provide practical benefits, the use of longer substrates more accurately mimics native mRNAs. Transcripts are exported from the nucleus with poly(A) tails of 70–80 adenosines in *S. cerevisiae* and 200–250 adenosines in higher eukaryotes [1]. Subsequent deadenylation in the cytoplasm results in average steady-state poly(A) tail lengths of ∼30 and ∼80 adenosines respectively, when total mRNA is examined [45]. The use of substrates in the *in vitro* assay with long poly(A) tails may therefore better resemble the entire deadenylation process that occurs in the cell. In addition, sequences that regulate deadenylation in *cis*, such as motifs recognized by Pumilio proteins, are often distributed throughout 3′ UTRs, and can be separated from the start of the poly(A) tail by hundreds of nucleotides [46]. For this reason, RNAs with longer upstream sequences and poly(A) tail lengths up to 80 adenosines have been prepared for use as deadenylation substrates [31], [47].

**Fig. 2:**
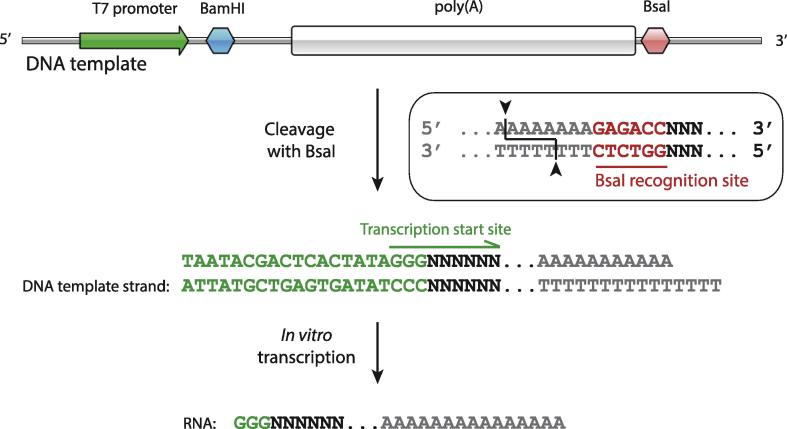
Preparation of an RNA substrate containing a poly(A) tail of uniform length. Schematic diagram of a DNA template used for transcription of poly(A)-containing RNAs. A restriction site (BamHI) located between the T7 promoter and a poly(A) sequence facilitates the cloning of any desired sequence to generate an upstream ‘UTR’-like sequence in the transcribed RNA. Cleavage of the template vector with the restriction enzyme BsaI yields a 3′ end that will terminate in poly(A) upon transcription.

Long RNAs are prepared by *in vitro* transcription reactions using T7 RNA polymerase and a DNA template encoding the desired RNA sequence [48], but it is technically challenging to prepare these with poly(A) tails of uniform length. The popular method of run-off transcription using linear DNA templates generated by standard restriction enzymes leaves non-adenosine nucleotides at the 3′ end. Amplification of a DNA template encoding a long poly(A) tail by PCR is also not possible, due to mis-priming on the homopolymeric poly(A) region. Below we outline strategies for generating RNAs with long poly(A) tails:

1. Poly(A) polymerase enzymes are commercially available, and can be used to add a non-templated poly(A) tail to RNA [49]. The length of the poly(A) tail can be controlled by judicious reaction conditions, and improved by gel purification. Still, the substrate is not entirely homogeneous in length, limiting precise quantitation of the reaction.
2. A defined RNA 3′ end can be generated after *in vitro* transcription using an encoded downstream ribozyme. While the more commonly used hammerhead ribozyme has a requirement for upstream sequence features that prevents its use in preparing terminal poly(A), the HDV ribozyme does not [50]. Use of this method has not, however, been reported for preparing RNAs encoding a poly(A) tail.
3. We use DNA templates terminating in an encoded 3′ poly(A) sequence generated from a plasmid treated with a Type IIS restriction enzyme (such as BsaI), which cleaves outside an asymmetric recognition site. This strategy yields RNAs with poly(A) tails of uniform length by *in vitro* transcription. First, a DNA plasmid containing a T7 RNA polymerase promoter and an encoded poly(A) sequence is made (Fig. 2). A restriction enzyme site between these sequences (e.g. BamHI) permits insertion of DNA of the desired sequence that will form part of the 3′ UTR in the RNA product. A BsaI restriction enzyme site is placed downstream of the encoded poly(A) tail such that cleavage occurs upstream of the recognition element, leaving terminal poly(T) on the template strand. Run-off transcription thereby generates an RNA with a poly(A) tail length equivalent to the length of the poly(T) sequence in the template strand (Fig. 2). Similar approaches have been reported using the restriction enzyme NsiI, which likewise generates a terminal thymidine on the template strand [51], [52], [53]. A HindIII sequence immediately adjacent to the poly(A) can also be used, with mung bean nuclease treatment employed to remove the single-stranded overhang [47].

Once the transcript is synthesized, it should be purified. This is best carried out by separating the desired transcript from other contaminating nucleic acids on a denaturing polyacrylamide gel, excising the band containing the desired transcript, and eluting the RNA, e.g. by electroelution.

While longer RNAs produced in this manner may be necessary for investigating certain processes, there are several limitations to their use. Firstly, run-off transcription reactions yield RNAs with heterogeneous 3′ ends, including truncated products with shorter poly(A) tails and RNAs containing non-templated terminal nucleotides [54]. The uniformity in RNA length is typically limited by the stringency of gel extraction performed after *in vitro* transcription. This heterogeneity in 3′ ends results in asynchronous reactions that are difficult to quantitate. A second major limitation of using long RNAs is their reduced resolution on denaturing gels: while shorter RNAs (≤50 nt) can be analyzed at single nucleotide resolution, longer RNAs require longer gels, lower percentage acrylamide and increased electrophoresis durations. All of these factors reduce the resolution that can be achieved. Thirdly, long UTRs have an increased propensity to become structured. Re-folding techniques such as heat treatment may be required to mimic structures within natural mRNAs, remove unwanted secondary structures, and achieve conformational homogeneity [55]. Finally, uridine-rich sequences within the UTR can base-pair with the poly(A) tail, and this can inhibit deadenylation [32].

### 3.3 RNA detection

The method of RNA visualization is another important consideration in the design of substrates for deadenylation reactions. Fluorophores can be incorporated at the 5′ end of short chemically synthesized RNAs. 5-carboxyfluorescein (5-FAM) or 6-carboxyfluorescein (6-FAM) are often used, and are available from many commercial suppliers. 3′ end-labeling or internal-labeling with fluorophores may interfere with the deadenylation reaction, and so should be avoided.

RNAs generated by *in vitro* transcription can be 5′ end-labelled with radioactive ATP(^32^P) using T4 polynucleotide kinase [56], or a fluorophore using maleimide-based conjugation chemistry [57]. Alternatively, a sensitive nucleic acid stain such as SYBR Green II can be used to detect unlabeled RNA [31]. Stains have the disadvantage that the signal correlates not only with the quantity of RNA, but also with RNA length. Shorter fragments generated by deadenylation will therefore have lower signal per molecule than the substrate, complicating quantitative analysis.

## 4 Design of deadenylation reaction conditions

### 4.1 Buffers

An optimal deadenylation reaction should include:

1. A buffer to maintain the pH between 6.5 and 8.5.
2. Monovalent salts. We have found that the activity of the Ccr4-Not complex is highly sensitive to salt concentration (see Section 8), and, like many other nuclease enzymes, is more active at low salt concentrations [58].
3. Magnesium. The activities of all major eukaryotic deadenylase enzymes (Ccr4, Caf1, Pan2, and PARN) are dependent on the presence of divalent cations, typically included at 1–2 mM as magnesium acetate or chloride salt [24], [32], [33], [39], [59], [60]. RNA is commonly stored in buffers containing EDTA – this needs to be taken into consideration because magnesium will be chelated by EDTA upon addition to the deadenylation reaction.
4. Reducing agents such as DTT and TCEP – typically at a concentration of 0.1–1 mM.

Other potential additives include detergents (e.g. Tween-20, NP-40), spermidine, BSA, and glycerol [15], [49], [61], [62]. These can act as molecular crowding agents and may increase protein solubility. While they may be essential if the enzyme is only available at low concentrations, we do not recommend their use as they may destabilize cytosolic protein complexes, and change the general properties of the reaction, complicating interpretations of quantitative measurements.

### 4.2 Deadenylation enzymes

The concentration of enzymes, and their abundance relative to that of the RNA substrate, must also be considered. We found that in the absence of additional factors, it takes approximately 30 min for Ccr4-Not (100 nM) to fully deadenylate A30 RNA (200 nM) in our chosen buffer conditions (see Section 7). The nuclease subunits in isolation from the complex are slower than the Ccr4-Not complex [32]. For this reason, it has been common practice to use isolated enzyme at concentrations greater than or equal to substrate in *in vitro* deadenylation reactions with recombinant Caf1 or Ccr4 [24], [25], [59]. Pan2-Pan3, however, is faster, and a molar excess of substrate can be used [31], [33].

The assays described here do not use a large excess of substrate over enzyme which would be required for determination of V_max_ and K_m_ using Michaelis-Menten models. The system as described here is set up to measure the effect of protein factors on complex deadenylation processes. Other types of assays, e.g. a direct readout of product release (AMP detection) at saturating RNA concentrations would be better suited for measurements of kinetic parameters.

### 4.3 RNA

The absolute quantity of RNA used in the reaction depends on the method of visualization. In general, 0.8 pmol of synthetic RNA labelled with 6-FAM provides adequate signal to measure the intensity of each RNA product band (see Section 7). Unlabeled RNA at a similar concentration can be detected using SYBR Green II stain. Radiolabeled RNA can be used at lower concentrations due to the higher sensitivity of detection [49]. This permits use of lower enzyme concentrations, which may be advantageous if protein availability is a limiting factor.

### 4.4 Accessory factors

Reaction conditions can be more difficult to optimize when additional protein factors such as Pab1 or RNA-binding adapter proteins are included in the reaction. The optimal conditions for these proteins may differ from those of the nuclease. We recommend first confirming the solubility of additional factors in the intended deadenylation reaction buffer, as well as their RNA-binding capacity. RNA-binding proteins commonly have off-target RNA-binding activity, so the concentration of each factor needs to be considered carefully. Furthermore, the biological relevance of the stoichiometry of RNA-binding protein to RNA should be evaluated. We recommend first characterizing RNA binding using a method such as electrophoretic mobility shift assay (EMSA) or fluorescence polarization. It may also be informative to perform a series of deadenylation reactions with varying concentrations of the accessory factor.

The sensitivity of the deadenylation rate to the concentration of monovalent salts makes it imperative that controls are performed appropriately, i.e. by adding an equal volume of storage buffer to negative control reactions. A difference in salt concentration as small as 10 mM could produce a change large enough to obscure the effect of the accessory factor (see Section 8).

Poly(A)-binding protein (Pab1/PABPC) binds to poly(A) RNA with high affinity [63], and promotes deadenylation by Pan2-Pan3 *in vitro* [31]. If the concentration of Pab1 protein were lower than that of the RNA, the RNA would not be fully bound and Pab1-bound molecules would be deadenylated at a different rate to the unbound molecules. In addition, Pab1 self-associates at high concentration [63], and binds non-poly(A) sequences [64] such as the upstream UTR of the deadenylation substrate. Therefore, the concentration of Pab1 protein should be equal or higher than that of the RNA, but large excesses should be avoided. We use a small molar excess of Pab1, for example 600 nM Pab1 and 180 nM A80-containing RNA [31]. Based on the low nanomolar published K_D_ values for the Pab1-RNA interaction, this should result in a fully bound poly(A) tail with up to three Pab1 molecules on each A80 tail [63]. This could be verified using EMSA on the deadenylation substrate.

## 5 Performing the deadenylation reaction

Deadenylation reactions are assembled as follows:

1. Mix the RNA with buffer solution at the desired concentration (e.g. 200 nM), but in the absence of Mg^2+^ to prevent metal-dependent hydrolysis. Heating the RNA-buffer mixture to 95 °C and cooling it rapidly on ice may be required to eliminate unwanted RNA secondary structure. Following cooling, the magnesium salt can be added to the desired concentration. An aliquot should be collected at this point to be run in the first lane of the gel. This serves as a control for the initial integrity of the RNA sample at time zero.
2. If additional factors such as Pab1 are to be tested, we recommend adding these to the RNA and allowing the binding equilibrium to be reached before the start of the reaction.
3. The reaction is initiated by the addition of enzyme at the desired concentration. We normally add 5 μl of Ccr4-Not at 1 μM to 45 μl RNA solution to obtain a final enzyme concentration of 100 nM. The reaction must be mixed thoroughly. Incomplete mixing can lead to high local concentrations of enzyme, and result in the rapid deadenylation of a subset of the RNA. This could be incorrectly interpreted as processive deadenylation. The reaction rate is temperature-dependent, and incubation is typically performed at 20–37 °C.
4. Aliquots of the reaction (e.g. 4 μl) are collected at a series of time points. By adding these to a solution of denaturing loading dye (2 × loading dye: 95% formamide, 10 mM EDTA, 0.01% w/v bromophenol blue), the reaction is stopped. We typically use 12 time points over 1–2 h to obtain enough data points for accurate quantification of deadenylation. Once added to loading dye, the samples can be briefly heated (95 °C, 2 min) to ensure complete protein denaturation and elimination of RNA structure. Samples can be analyzed immediately by gel electrophoresis (see Section 6) or stored at −20 °C.

Importantly, negative controls must be performed in parallel. These should include reactions where only the enzyme buffer and accessory proteins are added, and where a catalytically inactive mutant is used in place of the active deadenylase. This will confirm that the measured activity is solely due to the deadenylase enzymes and not a contaminating nuclease.

## 6 Electrophoresis and imaging

**Fig. 3:**
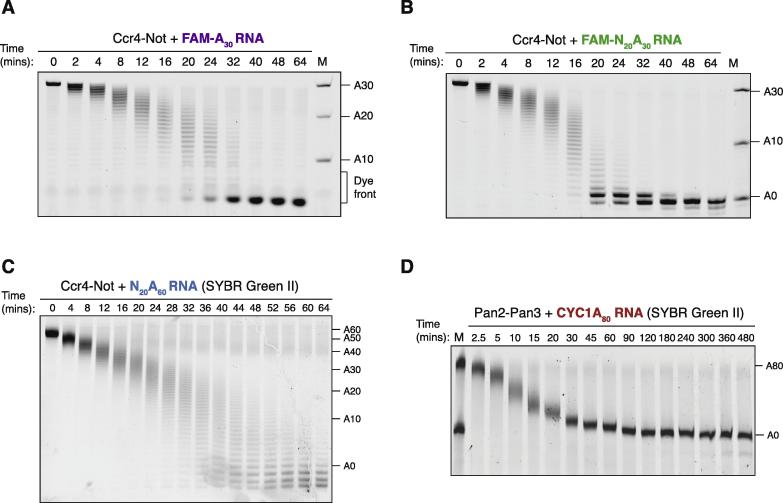
Visualization of deadenylation by denaturing PAGE. (A) Exonucleolytic degradation of a 30 nucleotide polyadenosine RNA by Ccr4-Not. Ccr4-Not (100 nM) was mixed with 5′ 6-FAM labelled A30 RNA (200 nM), and the reaction was analyzed at the indicated time points. RNA was resolved on a 20% acrylamide denaturing gel, and visualized by scanning the gel with laser excitation at 473 nm. (B) Deadenylation of a 5′ 6-FAM labelled RNA containing 20 non-poly(A) nucleotides upstream of a 30 nucleotide poly(A) tail (200 nM) by Ccr4-Not (100 nM). Reaction and visualization performed as in (A). (C) Deadenylation of an unlabeled RNA containing 20 non-poly(A) nucleotides upstream of a 60 nucleotide poly(A) tail (200 nM) by Ccr4-Not (200 nM). RNA was resolved on a 16% acrylamide denaturing gel. The gel was stained with SYBR Green II, and visualized by scanning the gel with laser excitation at 473 nm. (D) Deadenylation of a *CYC1* 3′ UTR (169 nucleotides; 180 nM) with an A80 tail by recombinant *Saccharomyces cerevisiae* Pan2-Pan3 (5 nM). RNA was resolved on an 8% acrylamide denaturing gel. The gel was stained with SYBR Green II as in (C). M is molecular weight marker showing poly(A) tail length including any upstream sequences. For (D), the upper band in lane M also acts as a 0 min time point.

Accurate quantification of the deadenylation rate can be achieved by separating the products of the reaction using denaturing PAGE (Fig. 3). Acrylamide gels are prepared at a percentage based on the separation range required (Table 1). Standard mini gels (∼6 cm) can resolve RNA substrates of less than 50 nucleotides at single-nucleotide resolution (Fig.3A, B). For example, deadenylation of RNA substrates with a 20 nt UTR and an A_30_ tail can be resolved to single nucleotides. While use of a longer gel improves the separation, short gels can be run in as little as 30 min, and are straightforward to prepare and handle. This makes them better suited to experiments performed with replicates, and to assess a wider range of assay conditions rapidly. Longer gels (∼15 cm) are needed to achieve single-nucleotide resolution for longer RNAs, greater than 50 nucleotides. Deadenylation of a substrate with an A_60_ tail is shown here as an example (Fig.3C).

**Table 1:** Percentage of acrylamide to use for various sized RNA substrates.

RNA substrate size (nucleotides)	Percentage acrylamide (w/v)
10	25%
10–50	20%
50–150	16%
≥150	6%

The acrylamide mixture should be prepared with 19:1 acrylamide/bis-acrylamide, 7 M urea and 1 × TBE buffer. The polymerization reaction is started by addition of ammonium persulfate (0.05% (w/v) final concentration) and TEMED (0.5 μl/ml of gel solution). Due to safety considerations, we recommend the use of commercially-available pre-made acrylamide solutions rather than making solutions from solid acrylamide. After polymerization, remove the comb, and wash all wells thoroughly with TBE running buffer using a syringe. This should be repeated immediately before samples are loaded for maximum resolution and to avoid streaking of the RNA caused by excess urea and unpolymerized acrylamide.

The gel can be pre-run for 20 min prior to loading, although this isn’t always required for short unstructured RNAs. Load the gel and include appropriate size markers. We prepare a control RNA lacking a poly(A) tail to define the anticipated end-point of the reaction. A size marker can be generated by alkaline hydrolysis of the substrate, which has the advantage of providing single-nucleotide resolution [65]. Run the gel at 400 V until the bromophenol blue dye front reaches the bottom. For a 20% acrylamide gel, the dye front indicates the location of RNA that is ≤8 nucleotides, which is the smallest product that can be quantitatively examined. Running the gel longer will further increase the separation of larger products.

Next, the gel is imaged. If the RNA substrate was labelled with a fluorophore, it can be scanned directly (e.g. using a Typhoon imager [GE]) after electrophoresis with fluorescence laser excitation at the appropriate wavelengths (for 6-FAM this is 470–490 nm). Gels containing RNA that is not labelled can be stained with SYBR Green II according to the manufacturers’ instructions. We find that it is important to wash the gels 5–10 times with 1 × TBE buffer after staining to minimize the background signal. Gels containing radiolabeled RNA are exposed to a PhosphorImager screen.

To maximize signal and avoid visible air bubbles, gels should be removed from the glass plates used for electrophoresis. Select a gain value that does not result in saturated pixels. If necessary, a neutral density filter can be used to dampen the signal and ensure detection is within the linear range of the detector. A complete absence of bands upon scanning indicates that too little RNA has been loaded on the gel, or the staining/phosphorimaging protocol has not been performed correctly. If RNA is only visible in the sample collected before addition of the enzyme, the enzyme may contain RNase contaminants that have digested the non-poly(A) portion of the RNA substrate. An RNA ladder can ensure that the separation range and gel running parameters are appropriate.

## 7 Analysis and quantification

In some cases, resolving the products of deadenylation at high resolution is not necessary: reaction rates can be estimated from the change in the poly(A) tail length without complete separation of RNA fragments if appropriate size markers are included on the gel (Fig.3D) [31]. Nevertheless, the use of high-resolution PAGE allows detailed interpretation of tail lengths at single nucleotide resolution. For example, a stretch of 14 uridines base-pair with a downstream poly(A) tail, and a footprint of 15 adenosines protected from the deadenylation activity of Ccr4-Not can be observed on the gel [32]. Analysis and quantification of poly(A) tail lengths can be carried out using ImageJ [66] or commercial software. Importantly, this type of quantitative analysis is limited to the particular buffer conditions, substrate concentrations, poly(A) tail lengths, and enzyme concentrations in the reaction. Thus, these analyses are best used for comparative measurements between different conditions.

**Fig. 4:**
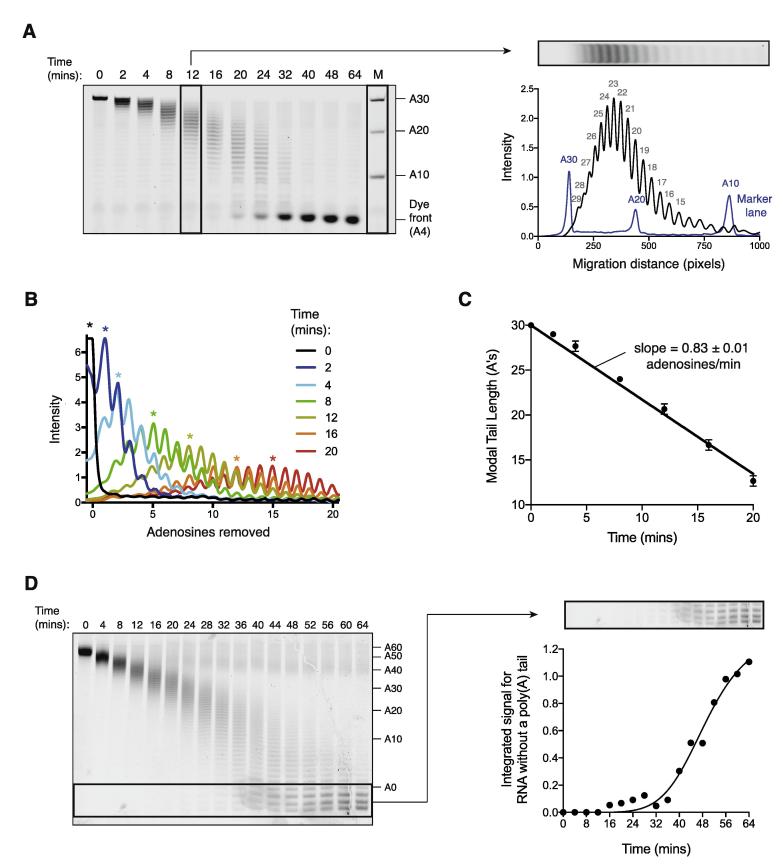
Analysis and quantification of gels. (A) Densitometric analysis of RNA separated by denaturing PAGE (gel from Fig.3A). Peaks in the black plot on the right correspond to RNA products in the indicated gel lane that differ in length by single adenosines. The most abundant RNA product (with modal poly(A) tail length) is 23 adenosines by reference to the markers of known length (blue). (B) Densitometric profiles of selected lanes of the gel shown in (A). A logarithmic transform was applied so RNA length is linear relative to the migration distance. Asterisks indicate the most abundant RNA products (modal poly(A) tail length) for each lane. (C) Plot showing the change in the modal poly(A) tail length with time. The average rate of deadenylation is the slope of the fit line. Error bars indicate the standard deviation in reaction triplicates. (D) Quantification of deadenylation rate determined by plotting the accumulation of fully deadenylated RNA with time. The signal intensity of bands representing RNA without a poly(A) tail on the gel (left, from Fig.3C) was integrated using ImageJ and plotted (right). The data were modeled with a sigmoidal function. (For interpretation of the references to colour in this figure legend, the reader is referred to the web version of this article.)

For reactions that proceed linearly, with no substantial changes in rate based on the length of the poly(A) tail, the average rate of the reaction can be obtained by plotting the size of the most abundant RNA species. We refer to this as the modal poly(A) tail length. To determine this, a densitometric profile of each lane of the gel is generated using the ‘Gels’ tool in ImageJ (Fig.4A). If the reaction products are visible at single-nucleotide resolution, the poly(A) tail length of the most prevalent RNA species can be counted, with reference to the RNA size markers. If the resolution of the gel does not allow RNAs differing in size by single nucleotides to be counted, the tail length can be estimated using size markers.

Importantly, the migration position of bands in electrophoretic gels do not linearly correlate with RNA size. While not essential to the calculation of the deadenylation rate, a plot can be generated that adjusts for this, and represents poly(A) tail length in a linear fashion (Fig.4B). To do so, a logarithmic transform is applied to displacement along the gel (in pixels), optimized to match the position to markers of known lengths.

Once the modal poly(A) tail length has been calculated for each time point, a plot can be generated for the length of the poly(A) tail with respect to time (Fig.4C). If appropriate, a linear fit can be applied to the data, where the average reaction rate is the slope (adenosines/min). The fully deadenylated RNA species will accumulate at later time points, and these should not be included in the linear fit. We find that the *in vitro* deadenylation reactions are highly reproducible, and the standard deviation of reaction rates calculated from triplicate experiments is typically low.

While plotting the modal poly(A) tail length works well for reactions that are distributive, it should not be used for highly processive reactions where the enzyme undergoes multiple turnover events without dissociating from the RNA. In this case, fully deadenylated RNA accumulates while RNA with an intact poly(A) tail is still present in the reaction. As a result, RNAs with intermediate tail lengths are not observed and deadenylation reactions can be analyzed by calculating the rate at which fully deadenylated RNA accumulates. First, invert the contrast to display white bands on a black background using ImageJ. Draw a box around the area of the gel that includes completely deadenylated RNA. It is important that this also includes all bands that correspond to RNA fragments generated by digestion of the non-poly(A) sequence, as shown in Fig.4D. Next, use the plot profile tool to generate a histogram in which a peak is displayed for each lane of the gel. The area under the peak represents the total amount of RNA that does not have a poly(A) tail, and this should increase with time. These values can be calculated using the tracing tool (wand). The integrated intensity of the zero time point should be subtracted from all measurements as it represents the background. Finally, generate a plot for the change in the integrated signal for RNA without a poly(A) tail with time (Fig. 4D). The time required for 50% of the RNA to be fully deadenylated can be derived from this plot and used as a metric of reaction rate.

Differentiating between distributive versus processive enzymatic activity is only valid under conditions when the substrate is saturating with respect to the enzyme. To achieve this, the substrate concentration must be significantly greater than enzyme concentration in the reaction mix.

To establish the statistical significance of assays, each should be performed at least in triplicate in order to determine the error related to the experiment, such as electrophoretic artifacts and the low signal to noise ratio in densitometric analyses. For each data point, error bars indicating standard deviation should be plotted. If a linear fit is applied, R-squared values for the best fit straight line can be provided. Residuals can also be plotted to ensure that a linear fit is justified. Finally, when comparing deadenylase activity under different conditions, such as in the presence of accessory factors, standard statistical analyses such as the two-sample *t*-test can be applied to test for statistical significance. These statistical tests should be used to compare a particular reaction condition with a negative control, e.g. a reaction where the accessory factor is added should be compared to a reaction where that factor is omitted (and buffer is added instead).

## 8 Optimization of the deadenylation reaction

The rate of *in vitro* deadenylation catalyzed by the Ccr4-Not and Pan2-Pan3 complexes depends on pH, magnesium concentration, salt concentration, and temperature. It has previously been reported that 0.1 mM magnesium yields highest activity for isolated Ccr4 from *S. cerevisiae* [23], but concentrations of 0.5–10 mM were found to produce the highest rates for the *H. sapiens* ortholog CNOT6L [24]. Manganese can be substituted for magnesium with isolated Caf1 [67], but it reduces the specificity of the enzyme for poly(A) [25], [68].

**Fig. 5:**
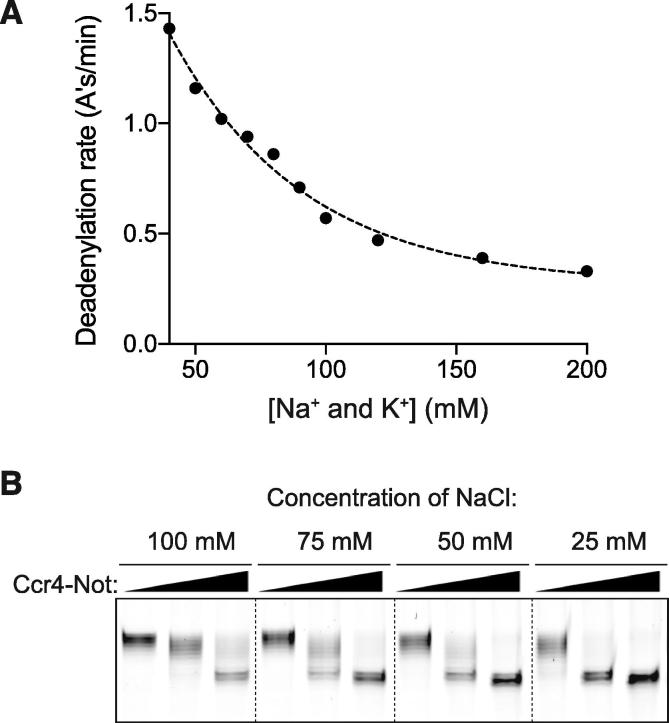
Optimization of *in vitro* Ccr4-Not reactions. (A) Relationship between deadenylation reaction rate and NaCl + KCl concentration. A series of reactions were performed on the A30 RNA with a titration of NaCl. 30 mM NaCl is carried over from purified Ccr4-Not stocks and deadenylation buffer contains 10 mM KCl. The total concentration of monovalent ions is plotted with respect to the reaction rate, determined as in Fig.4C. (B) Endpoint reactions: deadenylation reactions were performed with Ccr4-Not, an RNA containing 20 non-poly(A) nucleotides upstream of a 30 adenosine poly(A) tail, and varying concentrations of NaCl (100–25 mM). Reactions were stopped after 15 min by the addition of denaturing loading dye. Three concentrations of Ccr4-Not were tested for each concentration of NaCl (62.5 nM, 125 nM and 250 nM), with RNA at 200 nM.

As discussed in Section 4, the absolute and relative quantities of enzyme, RNA, and accessory proteins added to the reaction are important for interpretation. For this reason, it is important to optimize the reaction conditions using quantitative methods to evaluate changes in reaction rates as described in Section 7. For example, we tested the effect of NaCl on deadenylation assays with Ccr4-Not and an N_20_A_30_ RNA (Fig.3B, 5A). The deadenylation rate was quantified with a linear fit as shown in Fig.4C. The reaction rate decreases with increasing NaCl concentration (Fig.5A). Doubling the salt concentration from 50 mM to 100 mM caused an ∼twofold decrease in the reaction rate. For each NaCl concentration in Fig.5A, an entire time course is required. Alternatively, we have also used end-point reactions for a more qualitative optimization (Fig.5B). Deadenylation reactions were performed as above with various concentrations of NaCl, and the reaction was stopped after 15 min and analyzed by denaturing PAGE (Fig.5B). By resolving only a single time point (at a few different concentrations of enzyme), rather than a time series, more conditions can be tested in parallel.

## 9 Conclusions

In this report, we describe a PAGE-based method for characterizing deadenylation activity *in vitro*. A major advantage is that this technique is quantitative and also reveals the pattern of nuclease activity, i.e. relative processivity and degradation of poly(A) vs non-A upstream ‘UTR’ sequences. Other methods could be valuable for evaluating the kinetics of deadenylation, e.g. colorimetric detection of AMP release or FRET-based detection [69], but these do not allow the pattern of deadenylation to be observed.

A growing number of protein factors and RNA features have been implicated in the control of deadenylation, making validation of these findings and exploration of the mechanisms by which they occur of increasing importance. Our reaction setup allows the rigorous analysis of deadenylation systems with high temporal and nucleotide resolution. In the future, this *in vitro* system could also aid in the analysis of the activities of other RNase or DNase enzymes.
